# The Human Dynamic Clamp Reveals the Fronto-Parietal Network Linking Real-Time Social Coordination and Cognition

**DOI:** 10.1093/cercor/bhz308

**Published:** 2019-12-20

**Authors:** G Dumas, Q Moreau, E Tognoli, J A S Kelso

**Affiliations:** 1 Human Genetics and Cognitive Functions, Institut Pasteur, UMR3571 CNRS, Université de Paris, 75015 Paris, France; 2 Human Brain and Behavior Laboratory, Center for Complex Systems and Brain Sciences, Florida Atlantic University, Boca Raton, FL 33431, FL, USA; 3 Department of Psychology, Sapienza University, 00185 Rome, Italy; 4 IRCCS Fondazione Santa Lucia, 00100 Rome, Italy; 5 Intelligent Systems Research Centre, Ulster University, Derry, BT48 7JL, UK

**Keywords:** EEG, rTPJ, self–other integration, social cognition, virtual partner interaction

## Abstract

How does the brain allow us to interact with others? Social neuroscience has already provided some answers to these questions but has tended to treat high-level, cognitive interpretations of social behavior separately from the sensorimotor mechanisms upon which they rely. The goal here is to identify the underlying neural processes and mechanisms linking sensorimotor coordination and intention attribution. We combine the human dynamic clamp, a novel paradigm for studyingrealistic social behavior, with high-resolution electroencephalography. The collection of humanness and intention attribution reports, kinematics, and neural data affords an opportunity to relate brain activity to the ongoing social behavior. Behavioral results demonstrate that sensorimotor coordination influences the judgments of cooperativeness and humanness. Analysis of brain dynamics reveals two distinct networks related to the integration of visuo-motor information from self and other which overlap over the right parietal region. Furthermore, judgment of humanness and cooperation of others modulate the functional connectivity between this right parietal hub and the prefrontal cortex. These results reveal how distributed neural dynamics integrates information from “low-level” sensorimotor mechanisms and “high-level” social cognition to support the realistic social behaviors that play out in real time during interactive scenarios.

## Introduction

**Figure 1:**
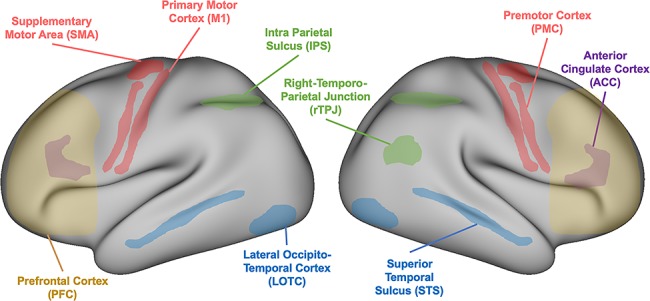
Schematic representation of brain areas associated with action perception, action execution, and reciprocal behaviors.

Much of our social life consists of interactions with others. Despite their essential role, social interactions still remain the “dark matter” of social neuroscience: the field is in urgent need of studies that embrace the reciprocal and real-time nature of social coordination (Hari and Kujala 2009; Dumas 2011; Hasson et al. 2012; Konvalinka and Roepstorff 2012; Schilbach et al. 2013; Hari et al. 2015). Overcoming the methodological challenge of bringing a true interactive context into the laboratory, some innovations have allowed behavioral tracking, brain recording, and stimulation of multiple participants in interaction (Montague et al. 2002; Babiloni et al. 2006; Tognoli et al. 2007; Dumas et al. 2010; Funane et al. 2011; Moreau et al. 2016; Chen et al. 2017; Dikker et al. 2017; Hirsch et al. 2017; Novembre et al. 2017; Era et al. 2018b). Such investigations have exposed the neural underpinnings of our propensity to interact with others during sensorimotor coordination (sensorimotor coordination is defined here as the coupling at the behavioral level through action-perception loops. In the present task, this is quantified by a phase relationship) (Tognoli and Kelso 2015). Functional neuroimaging studies have revealed several neural structures involved in simultaneous action perception, action execution, and reciprocal behaviors. A nonexclusive list includes visual cortices such as lateral occipito-temporal cortex (Lingnau and Downing 2015) and superior temporal sulcus (STS; Chauvigné et al. 2018), the parietal lobe including the intra parietal sulcus and the temporo-parietal junction (TPJ; Chauvigné et al. 2018), and frontal motor areas comprising the primary motor cortex (M1; Kilner et al. 2007), the premotor cortex, and the supplementary motor area, but also the anterior cingulate cortex (Apps et al. 2016) and the prefrontal cortex (Shaw et al. 2018; cf., Fig. 1).

At the neurophysiological level, several frequency bands have been implicated in social processes, including fronto-central and occipito-temporal Theta (4–7 Hz; Dumas et al. 2012; Moreau et al. 2018), fronto-parietal Alpha (8–13 Hz; Tognoli et al. 2007; Konvalinka et al. 2010; Novembre et al. 2014; Tognoli and Kelso 2015), rolandic Beta (Ménoret et al. 2014), and parietal Gamma (>30 Hz; Dumas et al. 2010, 2012).

Social neuroscience studies that investigate the interaction between multiple participants face the challenge of having more sources of unconstrained variance than experimental paradigms with single participants do. To overcome this limitation while at the same time maintaining the essential reciprocal and dynamical nature of social interaction, we use the human dynamic clamp (HDC) paradigm which enables direct experimental control of one of the social partners, as well as the coupling between them. The HDC consists of a human interacting reciprocally with a virtual partner (VP), the design of which is based on an empirically grounded computational model of human coordination dynamics (Kelso et al. 2009; Dumas et al. 2014a; Kostrubiec et al. 2015). This attempt to integrate empirically validated models is thus a departure from an ad hoc approach to modeling avatar behavior. Thus, the VP’s behavior is neither the product of a scripted scenario nor the sole outcome of the human’s behavioral dispositions, but rather a truly emergent collective pattern that results from their interaction. Both the intrinsic dynamics of the VP and its coupling to the human can be manipulated in real time thereby enabling the parametric exploration of the relationship between humans and interaction-capable surrogates. Analogous to its cellular counterpart (Prinz et al. 2004), the HDC paradigm offers a computationally precise way to approach the complexity of real-life social interaction while at the same time maximizing experimental control.

Divergent theories of social cognition have risen over the years. On the one hand, cognitive theories have focused on “top-down” processes (Sebanz et al. 2006), mentalizing and theory of mind (Frith and Frith 2012); on the other hand, sensorimotor theories have focused on the spontaneously self-organizing nature of coordination (Oullier et al. 2008; Coey et al. 2012), mirroring (Rizzolatti and Sinigaglia 2016), and sensorimotor coupling (Hari and Kujala 2009). The fully bidirectional nature of social behavior in terms of the perception-action has stressed the reciprocal relationship between levels of analysis and direction of information flow (Kelso et al. 2013). Previous behavioral studies have shown that whether humans perceive VP behavior as cooperative or competitive is modulated by coupling strength (coupling strength is a function of the parameters A and B in the HKB model. Drever et al. (2011) used β = 0.2 and 0.5, respectively, for weak and coupling strength) (Drever et al. 2011). Interestingly, however, automatic imitation processes (i.e., visuo-motor interference) seem to be present in both competitive and cooperative scenarios (Era et al. 2018a). Using the HDC, successful sensorimotor coupling (i.e., stably coordinated movements) and attribution of humanness to the VP have been associated with increased emotional arousal (Zhang et al. 2016). Moreover, under certain conditions, such as competing task requirements, humans spontaneously endow the VP with intention and goal-directedness (Kelso et al. 2009; Drever et al. 2011). This is in line with the broad literature on “intentional stance,” describing how humans have a seemingly irresistible disposition to attribute beliefs, desires, and intentions to the actions of others, including virtual agents (Gallagher et al. 2002; Dennett 1971).

The present work aims to elucidate so-called top-down and bottom-up perspectives of social behavior using real-time interaction between a human and a VP in conjunction with neurodynamical analyses of spatially resolved high-density electroencephalography (EEG) recordings. A benefit of using the HDC is that the evolving brain dynamics can be correlated with both human and VP movements as well as their coordination. In addition, the paradigm enables an evaluation of how humans reflect on their interaction with the VP and assess their (virtual) partner’s humanness and intention. The goal is to expose the brain circuitry hypothesized to relate real-time coordination and ongoing cognitive and emotional aspects of social behavior.

As noted above, various neurophysiological markers have been implicated in social processes. Accordingly, EEG analyses were conducted across all frequencies and without predetermined judgment regarding regions of interest. Via this strategy, we aimed to: 1) quantify how the dynamics of social coordination with a VP affects human subjective reports (cooperation ~ competition); 2) identify neuromarkers (local oscillations, transiently coupled functional networks) associated with sensorimotor coupling (i.e., integration of movements from self and other); and 3) investigate the neural correlates of humanness and intention attributed to a VP. Overall, our analyses and results at both neural and behavioral levels go some distance toward resolving previous antagonisms between online and offline modes of social cognition, and rather attest to their complementary nature.

## Materials and Methods

### Participants

About 20 volunteers, 12 males, and 8 females aged between 18 and 33 years (mean = 24.2, STD = 4.5) took part in the study. All were right-handed (Edinburgh Handedness Inventory) with reported normal or corrected-to-normal visual acuity and without self-reported history of neuropsychiatric disease or movement disorder. Participants provided informed consent prior to the research. The study was approved by the Internal Review Board at Florida Atlantic University and conformed to the principles expressed in the Declaration of Helsinki.

### Material and Apparatus

**Figure 2:**
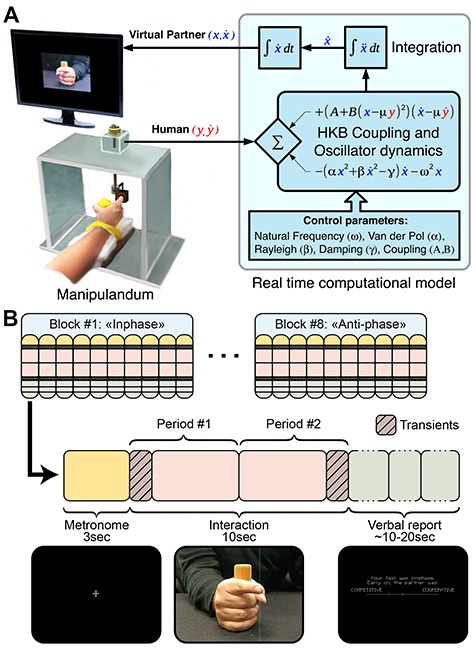
Schematic of the Human Dynamic Clamp paradigm. (*A*) Schematic of the HDC experimental apparatus. Human participants coordinate finger movement with a VP displayed on a screen. Movements are digitized and fed into a computer, where the HDC software computes in real time the corresponding position of the VP following the Haken-Kelso-Bunz (1985) model. The key parameters of the HKB model are the VP’s intrinsic frequency (ω = 1.6 Hz), and terms that control movement shape and various dynamical properties of the oscillator for behavioral realism (Van der Pol α = 0.641, Rayleigh β = 0.007095, and damping γ = 12.457), the coupling (A = −0.5 and B = −0.25), and the in-phase/anti-phase preference (μ = +/−1). (*B*) Summary of the experimental paradigm (a nonverbal Turing test) showing the structure of trial blocks (see text for details).

Participants were seated in a dark Faraday room, with the ulnar side of the right forearm resting against a U-shaped support (21.5 × 8 × 4 cm) positioned parallel to a table (Fig. 2*A*). Participants supported their right hand by grasping a vertical wooden cylinder (4.5 × 3 cm), leaving only the right index finger in extension. The hand was oriented in the sagittal plane, and the distal part of the index finger was inserted into the circular orifice (2-cm diameter) of another wooden block. The latter was connected through two metallic bars to a vertical, freely rotating metallic stem (18-cm length) whose angular displacement was captured by a linear potentiometer placed atop. The entire arrangement constituted a manipulandum, which was fixed on the top of a Plexiglas box (30.5 × 31.5 × 20 cm), positioned to the right of a screen, about 50 cm away from the midline of the participant. The manipulandum restricted the movement of the index finger to the horizontal plane and allowed a full-range of friction-free flexion-extension motion about the metacarpophalangeal joint.

The output of the potentiometer was sampled at 1000 Hz in step with the EEG, using a National Instruments A/D converter. The signal was down-sampled offline to 500 Hz for online computational efficiency, and used as a continuous input into a computer, which ran the HDC program implemented on C++ using the cross-platform IDE Code Blocks and the open source library OpenFrameworks (code available at https://github.com/GHFC/SoNeTAA). A java script version of the HDC is available on GitHub: https://github.com/crowd-coordination/web-vpi and can be tested online at http://www.morphomemetic.org/vpi/. The velocity of the human finger was numerically computed using a three-point differentiation algorithm and together with position data plugged into the VP equation (cf., Dumas et al. 2014a for details). The differential equation returned instantaneous VP acceleration, which was integrated using a fourth-order Runge–Kutta method at 500 Hz to provide VP velocity and position. We compared digital timestamps and analog triggers with an oscilloscope to ensure that a maximum delay of 2 ms occurred between data acquisition and computation of the model output (0.3% of typical movement cycle length).

To create the animation of VP finger movement used in the experiment, a series of position-indexed images was created and stored (cf., Kelso et al. 2009). The images were recorded with a high-speed camera while a human male hand produced flexion-extension finger motions in the horizontal plane. A complete cycle of movement provided 119 images (17 × 13 cm) indexed by their position. The instantaneous position of the VP, as computed in real time during the experiment, was iteratively used to select one of the 119 position-indexed images, which was displayed in the center of the screen (59-cm diagonal). The screen animation was refreshed at 100 Hz during the experiment and looked just like an ordinary video display of a real finger in repetitive motion. An auditory tone of 440 Hz and 0.1 s duration was used as a pacing signal to constrain the initial frequency difference between human and VP and to minimize intertrial and intersubject variability accordingly.

### Experimental Design

Participants interacted with the VP during a session composed of 80 pseudo-randomized trials (Fig. 2*B*). During the instruction phase, the experimenter demonstrated the ability of a second manipulandum, placed in participant’s view just outside the EEG chamber, to provide real-time control of finger movement. While demonstrating the ability of the second manipulandum to control the VP on the participants’ screen, the experimenter told a cover story pretending that the VP will sometimes be controlled by another participant (even if it was all the time controlled by a computational model). The addition of this manipulandum not only assisted in conveying task instructions but also provided a basis for the participant to attribute observed finger movements to another human. Trials were arranged in blocks of 10 trials, during which participants were tasked to coordinate their movement either in-phase or anti-phase with the VP. The order of blocks was counterbalanced across participants.

Each trial was composed of three periods (Fig. 2*B*): pacing (3 s), interaction (10s), and self-report. Participants were instructed to maintain coordination with the VP during the interaction according to block-wise instructions (i.e., in-phase or anti-phase). Before each trial, a brief screen presentation indicated the instruction of the current block followed by a fixation-cross at the center of the screen. Then, the pacing period started, and an auditory tone cued the required movement frequency (1.6 Hz). Participants were instructed to produce peak flexion on each beat while fixating upon the cross and then to maintain the frequency throughout the rest of the trial. As soon as the pacing signal was turned off, the interaction phase began, and the moving VP finger appeared on the screen. During the interaction phase, participants were instructed to continue their finger movement while coordinating in-phase or anti-phase with the VP finger. The VP also had an intrinsic frequency of 1.6 Hz and a phase randomized and locked relative to the auditory metronome. The VP was randomly assigned a cooperative or competitive behavior for two halves of each trial (i.e., 4 s subperiod), giving four pseudo-randomized types of trials: cooperation throughout, competition throughout, switch from cooperation to competition, and finally switch from competition to cooperation. This cooperative/competitive behavior was parameterized using the in-phase/anti-phase preference (μ = +/−1), thus creating either VP’s sharing or antagonism to the participant’s goal. After the interaction period, the participants were reminded of the Task instruction (“your task was in-phase.” or “your task was anti-phase.”). Furthermore, they were asked to judge the VP using rating scales presented on the screen. In details, participants reported the degree to which the VP was cooperative or competitive early and later on during the trial (questions: “early on, the partner was …?” and “then, later on, the partner was …?”; continuous value from 0: competitive to 1: cooperative). At the end, they judged the humanness of the VP (question: “overall, how was the partner like?”; binary choice with 0: machine and 1: human). All reports were done using a scale on the screen which was controllable by the finger motion of participants. Since the resulting values were in a limited range and non-Gaussian, such data were analyzed by means of a Wilcoxon rank test. After each choice was dialed on the manipulandum, participants employed a short acquiescing sound to signal validation of their selection without talking, and the experimenter moved the program to the next trial. The nasopharyngeal sound (resembling/Ͻ/but without the labial component) had previously been established in pilot experiments to minimize muscular and movement artifacts at the sites of EEG recording.

### Behavior Analysis

Potentiometer signals corresponding to human finger displacement and position of the VP’s finger were mean-centered, detrended, low-pass filtered using a second-order dual-pass Butterworth with a cutoff frequency of 20 Hz and normalized. After this preprocessing, we quantified the coordination between human and VP by calculating the continuous relative phase (RP) between their movements (phase estimated with continuous Hilbert transform). To avoid transients and thus separate the inhomogeneous neural activity occurring at onset and offset of the interaction, we removed the first and last seconds of interaction, leaving 8 s of each trial for analysis. For each of the two halves of the trials, mean RP and the corresponding circular variability were calculated, in order to assess the produced pattern (e.g., in-phase, anti-phase, or some other pattern) and its stability, respectively. For each participant, subjective reports of humanness and cooperativeness were Z-score normalized, and performances were quantified through two indices: a phase coordination score equal to the normalized absolute difference between ongoing RP and the RP corresponding to the task condition (i.e., 0 rad for in-phase and pi rad for anti-phase); an intention attribution score equal to the normalized difference between perceived cooperativeness and real VP behavior, thus quantifying if the human participant was able to perceive VP’s helpfulness (or not) toward achieving his/her goals. Behavioral variables were analyzed through a repeated measures 2 × 2 × 2 × 2 ANOVA in JASP (JASP Team 2018) having as factors Trial-Part (First-Half/Second-Half), Behavior of the VP (Cooperative/Competitive), Transition during the trial (Yes/No), and Task (in-phase or anti-phase). Results were corrected for multiple comparisons using Bonferroni tests (significance level at *P <* 0.05).

### High-Density EEG Recording

The experiment was conducted in a sound-proof Faraday chamber. High-density EEG was recorded using 128 channel EEG caps with Ag-AgCl electrodes (Falk Minow Services) arranged according to an extension of the 10–20 system (Jasper 1958; Oostenveld and Praamstra 2001). The signals were fed to an amplifier (Synamp2; Neuroscan) equipped with high-level port to ensure the recording of triggers from the HDC software and to obtain temporally precise analysis of brain-behavior dynamics. EEG signals were measured with the respective ground located on the left shoulder and referenced at the Cz electrode. Impedances were maintained below 10 k∧. The signals were analog filtered (Butterworth, band pass from 0.05 Hz (−12 dB per octave) to 200 Hz (−24 dB per octave)), amplified (gain of 2010), and digitized at 1000 Hz with a 24-bit ADC in the range ±950 μV (vertical resolution of 0.11 nV).

### Skin Potential Response

Emotional arousal was quantified with a bipolar montage of two passive Ag/AgCl electrodes capturing sympathetic changes (one on the left palm and one on the left epicondyle as a reference, both placed on the immobile hand). We extracted the skin potential response (SPR) normalized magnitude by following the method described in Zhang et al. (2016) (where the main results from this experiment with respect to emotional arousal are reported).

### Artifact Correction and Data Preprocessing

Following visual inspection, any noisy EEG channel was marked as bad (average = 4.5, min = 0, max = 10) and interpolated using a spherical spline algorithm (Perrin et al. 1989) with an interpolation order *m* = 3, a Legendre polynomial order *n* = 50, and a regularization parameter λ = 10e−8 (Kang et al. 2015). Correction of eye blink artifacts in the EEG data was performed using a classical PCA filtering algorithm (Wallstrom et al. 2004) on 800-ms windows with 400-ms overlap. A Hamming window was used to control for artifacts resulting from data splicing. EEG signals were then visually checked to exclude from analysis all trials contaminated by residual eye blinks, unwanted swallowing, coughing, or movement artifacts. Following correction, EEG data were re-referenced to a common average reference. More than 70 artifact-free trials were obtained for all participants, and there were no differences in the overall quality of the data and the number of unrejected trials per condition.

### Single-Trial EEG Source Estimation

Source reconstruction was performed with the free open-source application Brainstorm (http://neuroimage.usc.edu/brainstorm; Tadel et al. 2011). Sensors were registered for each participant using head points and fiducial landmarks (nasion and preauricular points) digitized with a Polhemus Isotrak system and projected on the scalp surface of the standard Montreal Neurological Institute (MNI) template space (Holmes et al. 1998). The lead field was then computed using a boundary element model in OpenMEEG (BEM) (Kybic et al. 2005; Gramfort et al. 2010) with a cortical surface tessellated with 2000 vertices. A noise covariance matrix was estimated from a 2-min resting state condition. The inverse solution was estimated for each individual using a standardized low-resolution brain electromagnetic tomography method (sLORETA) (Pascual-Marqui 2002) with unconstrained source orientation. Thus, cortical sources were estimated at each vertex of the cortex surface with three orthogonal dipolar sources.

### Brain Analyses

The estimated cortical source dynamics was then processed for each trial by taking the interaction period without the first and last 1 s transients, since brain activity was expected to be nonstationary near these boundaries. The remaining 8 s were tapered with a 1-s Hamming window, and discrete Fourier transforms used to estimate spectra at the source level. We used Student *t*-test with false discovery rate correction for the statistical analyses of power modulations. Reports of anatomical regions followed the Tzourio-Mazoyer atlas (Tzourio-Mazoyer et al. 2002).

For functional connectivity, following Maris et al. (2007), the coherence between all pairs of sources was calculated for frequency bands of interest (cf., Table 1) according to the formula:

(1) \begin{equation*} {\mathrm{COH}}_{i,j}(f)=\frac{{\mathrm{s}}_{i,j}(f)}{\sqrt{{\mathrm{s}}_{i,i}(f)\mathrm{s}j,j(f)}}\quad \mathrm{With}\;{\mathrm{S}}_{i,j}(f)=\frac{1}{N}{\sum}_{n=1}^N{F}_i(f){F}_j(f), \end{equation*}

**Table 1:** Frequency bands of interest with their respective lower and higher bounds. All values in Hz.

Frequency band	Lower bound	Higher bound
F0 (fundamental of the movement frequency ± 1 Hz)	0.6	2.6
F1 (1st harmonics of the movement frequency ± 1 Hz)	2.2	4.2
Delta	1	4
Theta	4	7
Low-alpha	7	10
High-alpha	10	13
Beta	13	30
Gamma	30	60

where $i$ and $j$ are the sources index, ${\mathrm{S}}_{i,j}(f)$ is the cross-spectrum, $N$ is the number of windows (i.e., equals to 8), ${F}_i(f)$ is the complex Fourier component at frequency $f$ for source $i$, and denotes complex conjugate.

We then averaged the power and coherence values across trials and eliminated potential statistical bias due to the non-Gaussian distribution of coherence values and unequal sample sizes by using Z-Coherence (Maris et al. 2007).

The Z-Coherence is defined as:

(2) \begin{equation*} {\mathrm{Z}}_{i,j}(f)=\frac{\left(\left(\left|{\mathrm{COH}}_{i,j}^A\ (f)\right|\right)\!-\!\left(\frac{1}{df_A}-2\right)\ \right)-\left(\left(\left|{\mathrm{COH}}_{i,j}^B\ (f)\right|\right)\!-\!\left(\frac{1}{df_B}-2\right)\ \right)}{\sqrt{\left(\frac{1}{df_A}-2\right)+\left(\frac{1}{df_B}-2\right)}}, \end{equation*}

where ${df}_A$ and ${df}_B$ are the degrees of freedom in conditions A and B, and ${\mathrm{COH}}_{i,j}^A\ (f)$ and ${\mathrm{COH}}_{i,j}^B(f)$ are coherences in conditions A and B between sources $i$ and $j$ at frequency $f$. The sign of Z indicates whether coherence in condition A is higher (positive) or lower (negative) than in condition B. To target large-scale brain dynamics links with high-level cognition, we tested whether attribution of humanness and cooperative/competitive actions from the VP were associated with whole-brain network connectivity variations.

### Brain-Behavior Analyses

We also computed coherence between movement velocity and reconstructed cortical sources. In this case, the formula described for neural coherence in equation (1) still holds except that the source $j$ is not another cortical source but either the human or the VP movement velocity. To compute the Z-Coherence, we used as control condition (i.e., condition B, in equation (2), a phase scrambled behavior. We averaged the coherence maps over participants and contrasted the two conditions.

### Statistical Analyses

Student *t*-tests were computed for both power and Z-Coherence comparisons. All difference maps were thresholded at *P* < 0.05 by using group-level (*n* = 20), two-tailed paired permutation tests, which randomly exchanged the estimated values of coupling between conditions for each participant. We used exhaustive permutations (2^20) to estimate the empirical distribution under the null hypothesis of no difference between the two conditions (Pantazis et al. 2005; Maris and Oostenveld 2007). For cluster-based statistics, the connectivity matrix across the vertices was extracted from the BrainStorm MNI mesh previously used for source reconstruction analysis (i.e., vertices that are part of the same face of the mesh are considered as neighbors). The alpha level was adjusted to the maximum statistical distribution to control for type I family-wise error rate due to multiple comparisons over the entire brain surface. Unless otherwise stated, all coherence and power plots presented here were obtained with group-level (*n* = 20) statistical inference based on paired permutation tests. All randomizations were done for the rejection of the null hypothesis and to control the family-wise error rate at *P* = 0.05.

## Results

### Behavior

Behavioral analysis focused on subjective reports (accuracy in cooperative or competitive intention attribution) and coordination measures (stability of the RP during the interaction).

### Participants Accurately Judged Partner’s Intention

During the coordination task, both partners had to settle on a common frequency while being instructed to coordinate with each other either in-phase or anti-phase, two modes known to be stable (Haken et al. 1985; Kelso 1984). In agreement with an experimentally chosen parameter of strong coupling from the VP, the RP was mostly stable and followed the intention of VP.

**Figure 3:**
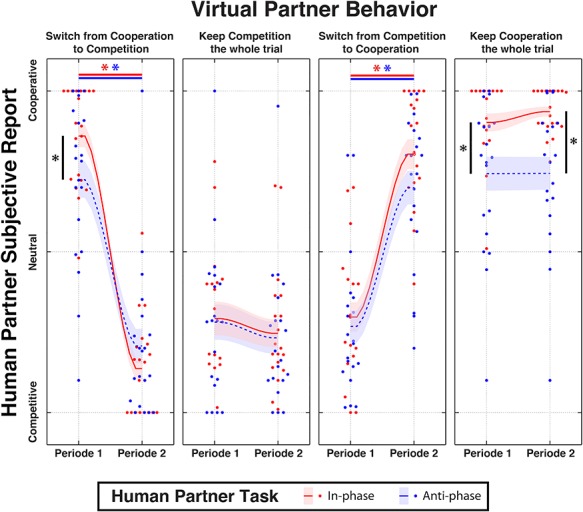
Judgment of cooperation and competition between human and VP. Subjective reports show how participants perceived the VP across the different conditions. Red and blue plots represent, respectively, the in-phase and anti-phase tasks, shaded areas represent standard errors, and stars indicate statistically significant differences (*P <* 0.05, corrected). See details in text.

After each trial, participants were asked to judge the intention of the partner, that is, was (s)he cooperative or competitive (cf., Fig. 3). Overall, they correctly attributed VP’s intention 80.3% of the time (*P* < 0.001, permutation test against chance; 10.6% false-cooperation, 9.1% false-competition), and their subjective reports were successfully modulated by the behavior of the VP during the interaction. The 2 × 2 × 2 repeated-measures ANOVA (VP Behavior [Cooperative/Competitive] by Transition [Yes/No] by Task [In-phase/Anti-phase]) on subjective reports of cooperativeness revealed a main effect of VP behavior (*F*(1, 19) = 579.49, *P* < 0.001, ηp^2^ = 0.650), showing that the VP was rated more cooperative in the cooperative condition compared with the competitive one. The transition factor also reached significance (*F*(1, 19) = 4.25, *P* = 0.04, ηp^2^ = 0.013), showing that a change in VP behavior during the trial resulted in the VP being rated less cooperative. Finally, there was a main effect of Task (*F*(1, 19) = 9.71, *P* = 0.002, ηp^2^ = 0.030), as well as an interaction between VP behavior and Task (*F*(1, 19) = 10.80, *P* = 0.001, ηp^2^ = 0.033). Post-hoc tests revealed that VP was judged less cooperative for the task anti-phase than in-phase (*t*(19) = −3.4, *P* < 0.001), especially when the trials started with a cooperative VP (Fig. 3).

One notable exception occurred when the human task to move in-phase led to a VP deemed less cooperative than when the human was tasked to move anti-phase (Fig. 3, left panel). This happened during the second part of trials with a competitive VP intending anti-phase, following initial segments when VP co-operated in-phase (“treachery”). In such cases, VP was deemed the most competitive of all (Fig. 3, left panel). Overall, the results already suggest that sensorimotor coupling and intention attribution are linked: the better able participants were in coordinating together, the more aware they were of their partner’s intention.

### Stability of VP Intention and Coordinative Stability Modulate Social Cognition

The 2 × 2 × 2 × 2 repeated-measures ANOVA (VP Behavior [Cooperative/Competitive] by Transition [Yes/No] by Task [In-phase/Anti-phase] by Trial-Part [First-Half/Second-Half]) on RP stability revealed that coordinative stability depended: 1) on the cooperativeness of the VP (*F*(1, 19) = 42.45, *P* < 0.001, ηp^2^ = 0.123) with competitive being less stable than cooperative behavior; 2) on the organization of the trial, with the first half of the trial less stable than the second (*F*(1, 19) = 15.97, *P* < 0.001, ηp^2^ = 0.050); and 3) on the presence of a transition in VP behavior, with the presence of a transition resulting in less stability (*F*(1, 19) = 19.34, *P* < 0.001, ηp^2^ = 0.060). The transition × trial-part interaction (*F*(1, 19) = 20.47, *P* < 0.001, ηp^2^ = 0.063) indicated that RP stability was lowest in the second half of the trials, in cases where VP switched behavior at mid-trial (*ps <* 0.001). Note that in all cases, stability was assessed in each half of the trial and after a transient to discard effects from the appearance of the partner and a switch in VP intention. Interestingly, accurate intention attribution was correlated with the stability of the interaction (*r* = 0.51, *P* = 0.02), pointing toward a link between the pattern of sensorimotor coordination and socio-cognitive assessment of the other.

### Cooperative VPs Were Judged More Human

**Figure 4:**
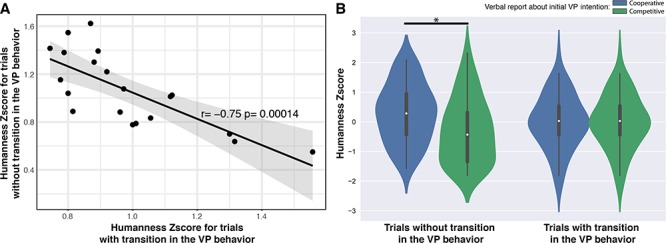
Humanness assessment during the interaction with the VP. (*A*) Transitions in VP behavior modulated the subjective feeling of interaction with another human, measured by normalized Humanness score (Z-scores, raw values were between 0 and 1, respectively, corresponding to the judgment of VP as a Robot or a Human). Shaded area indicates 95% confidence interval. (*B*) Modulation of perceived humanness by the presence of transition in VP behavior and its initially perceived cooperativeness. Star indicates statistically significant differences (*P* < 0.05, corrected).

Consistent with earlier reports that endowed VPs with intentionality (perceived as the VP trying to trick the human to achieve goals opposite to his/her, Kelso et al. 2009), we found that (objectively) cooperative VPs scored higher in humanness ratings than competitive ones (Wilcoxon rank test *Z* = 2.62, *P* = 0.008). Despite this prominent dependency on objective variables of cooperativeness, the humanness rating was independent of subjective judgment of cooperation. Indeed, subjective ratings of cooperativeness in both periods of interaction did not show any differences between trials associated with judgments of humanness (Wilcoxon rank test with *Z* = −0.53, *P* = 0.6 for the first part of the trial, and *Z* = −0.20, *P* = 0.84 for the last). Similarly, trials with both parts judged as cooperative did not demonstrate differences in humanness rating compared with trials with both parts judged as competitive (Wilcoxon rank test, *Z* = −1.5, *P* = 0.13). In contrast to intention attribution, judgment of humanness did not depend on RP stability (Wilcoxon rank test, *Z* = 0.64, *P* = 0.52). However, statistical analysis revealed a significant correlation between VP behavior and transition in the judgment of humanness (Fig. 4*A*), indicating that the latter depends on both cooperative behavior and how this behavior changes over time.

The 2 × 2 × 2 (VP Behavior [Cooperative/Competitive] by Transition [Yes/No] by Task [In-phase/Anti-phase]) repeated-measures ANOVA on humanness ratings showed a main effect of VP behavior (*F*(1, 19) = 7.022, *P* = 0.008, ηp^2^ = 0.022) as well as an interaction between VP behavior and transition (*F*(1, 19) = 7.022, *P* = 0.008, ηp^2^ = 0.022), suggesting that a drop in humanness rating occurred when VPs were acting in a competitive fashion and, specifically, when VPs sustained their competitive intention throughout the trial (cf., Fig. 4*B*).

### Right Parietal Activity during Human ~ VP Coordination

**Figure 5:**
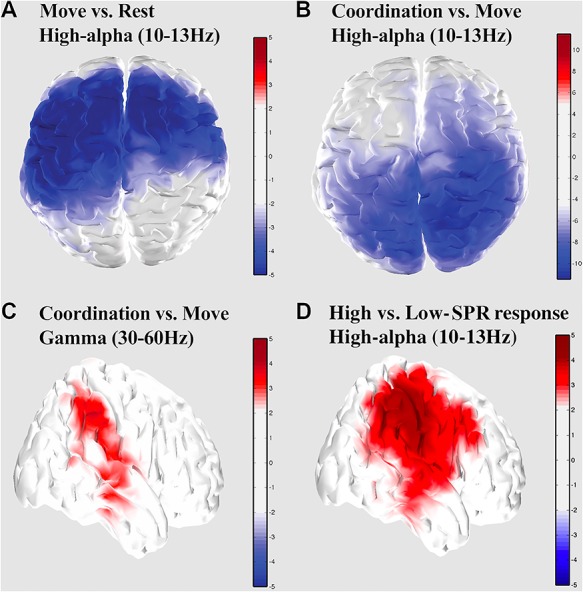
Modulation of spectral activity of cortical sources. High-alpha suppression over motor regions during the execution of movement without VP (*A*), joint decrease of high-alpha activity (*B*) and increase of gamma activity (*C*) over right parietal cortex during interaction with VP, and modulation of high-alpha activity by the emotional responses of the participants as measured by SPR (*D*). Color indicates clusters of cortical sources which were significantly modulated in each contrast.

Power analyses on estimated cortical sources revealed well-known mu suppression in the upper alpha band over primary motor areas during movement (maximum difference over precentral left with *t*(19) = −5.58, *P* < 0.0001; Fig. 5*A*) and also revealed a joint suppression of high-alpha band (maximum difference over parietal superior right: *t*(19) = −10.38, *P* < 0.001; Fig. 5*B*) and an increase in gamma band activity over right posterior cortex during active coordination with VP (maximum difference over temporal superior right: *t*(19) = 2.95, *P* < 0.05; Fig. 5*C*). Interestingly, the emotional response of participants (as quantified by SPR) was also linked to a strong modulation of high-alpha activity in the same anatomical regions (maximum difference over supra marginal right: *t*(19) = 3.55, *P* < 0.05; Fig. 5*D*).

### Neural Dynamics Coordinated with Self- and Other Behavior

**Figure 6:**
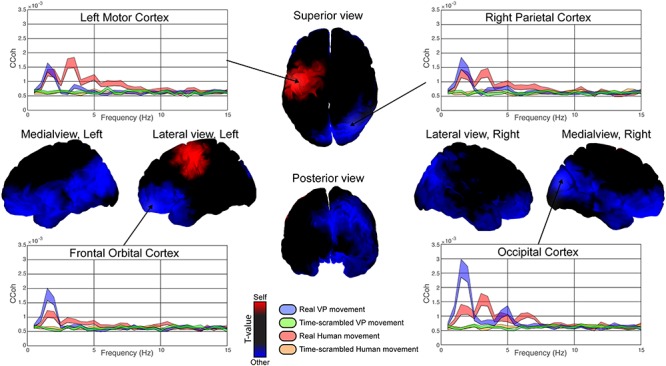
Self- and other-related cortico-motor coherence modulation. Graphs show cortico-motor coherence (Jerbi et al. 2007) across frequencies for key task-related brain structures. 3D brain figures represent statistical differences between cortico-motor coherence of human and VP movement velocities and neural activity in the delta/theta band (1–5 Hz). Red and blue indicate sources which were statistically more associated with (respectively) self- or other movement. Notice the increase for self-movement in the left motor cortex and for other movement in right parieto-occipital, left frontal and midline regions. Notice also the absence of coherence with time-scrambled movements (green and orange in the graphs). Shaded areas in 2D graphs represent standard errors.

Analysis of cortico-motor coherence with velocity of human movement (“self”) revealed the significant involvement of contralateral primary motor cortex (red colored region of the brain, maximum difference over precentral left: *t*(19) = 3.53, ***P*** < 0.005; Fig. 6). In contrast, when cortico-motor coherence was based on VP (“other”) velocity, an antero-posterior network was observed (blue colored regions of the brain, maximum difference over right cuneus: *t*(19) = 4.56, ***P*** < 0.0001; Fig. 6). Both brain networks, for self and other, span the fundamental frequency of movement and the first 2 or 3 harmonics (see distribution in the insert spectra, Fig. 6), roughly corresponding to the delta/theta bands.

**Figure 7:**
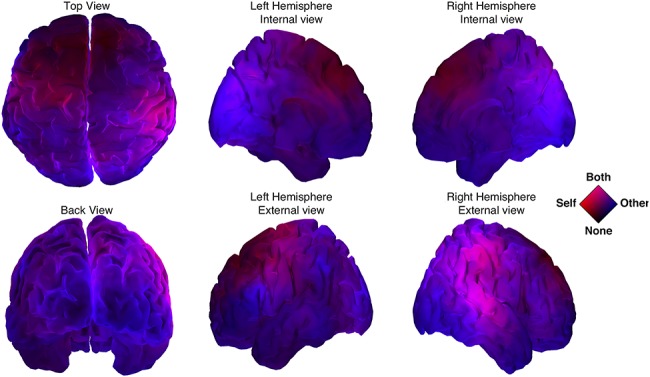
Overlap of self- and other-related brain networks. The networks related to self- and other-movement overlap on both rTPJ and right STS. Color stands for significance (−log(p)) of theta cortico-motor coherence contrasts (*P <* 0.05) for Real versus Scrambled behavior. Red and blue colors component code, respectively, for the “Self- versus Scrambled” and “Other- versus Scrambled” contrasts. We attract the attention to the bright magenta area which corresponds to cortical sources related to both Self- and Other behaviors.

Besides pointing to significant differences in corticomotor coherence between brain regions associated with self- and other movement, we can investigate how the two networks are integrated via an analysis of their overlap (Fig. 7). Joint self- and other-movement coherence activities were found over right parietal areas (purple) with four ROIs: cuneus right, angular right, parietal inferior right, and supra-marginal right (Fig. 7, pink color). Very few parts of the cortex were indifferent to both self and other’s movement (black color, restricted to superior frontal areas). The region that was specific for self-movement, again, was the contralateral motor cortex (red color). In contrast, a large expanse of the cortex was concerned with the other’s movement (blue color).

### Modulation of Functional Connectivity by Attribution of Humanness and Cooperation

**Figure 8:**
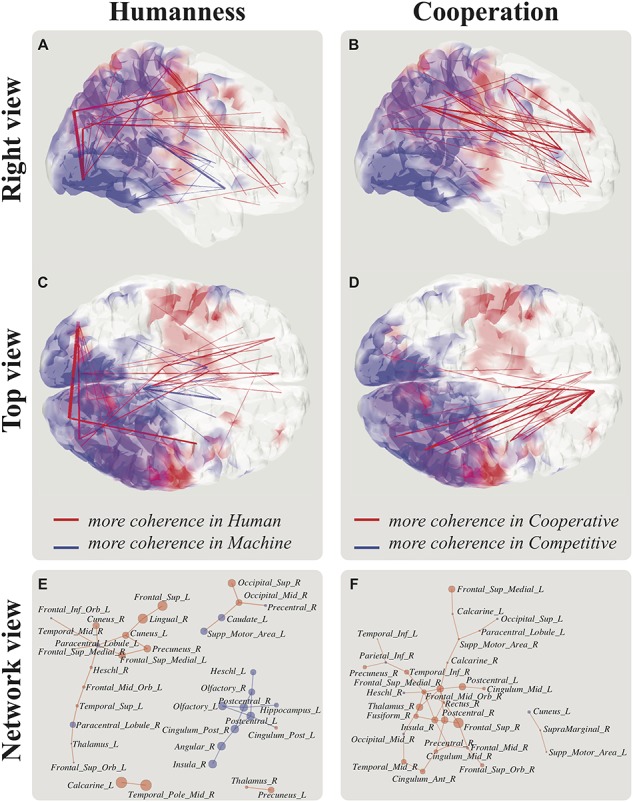
Right parietal cortex as a hub between self- and other-sensorimotor brain networks. Humanness attribution and cooperation are related to changes in large-scale brain dynamics. Coherence between posterior and anterior brain structures in the delta/theta band (1–5 Hz) increased when participants judged the VP as human rather than machine (*A*, *C*, *P* < 0.05 noncorrected) and when VP was cooperative rather than competitive (*B*, *D*, *P* < 0.05 noncorrected). Panels (*E*) and (*F*) show, respectively, the networks of brain structures implicated in (*A*, *C*) and (*B*, *D*). Line width and circle size indicate the modulation strength of coherence and power in the theta band.

Functional connectivity in the low-theta band was explored in order to understand how self- and other-related information may be related to cooperative/competitive behaviors or humanness judgment during social interaction. The two contrasts revealed a similar pattern: sensorimotor hubs in the posterior part of the brain, predominantly in the right hemisphere (e.g., Cuneus), were coordinated with anterior areas (e.g., Frontal Superior). Figure 8*A*–*D* illustrates the anatomical distribution of the most important changes due to coupling, revealing that both the attribution of humanness to the VP and cooperation of VP are associated with a coherence increase between posterior and anterior brain structures in the delta/theta band (1–5 Hz). Figure 8*E*,*F* provides further details on the brain areas involved in this increase (or decrease) of coherence according to the Tzourio-Mazoyer atlas.

## Discussion

The present study used the HDC to investigate the neural underpinnings of social interaction and in particular how the brain integrates its own behavior with that of others to support the attribution of humanness and intention. We recorded high-density EEG in humans interacting with a VP parameterized to act in a cooperative or a competitive way. At the behavioral level, our results suggest a link between sensorimotor coupling and intention attribution as shown by the correlation between individual phase coordination scores (stability) and accuracy in detecting VP’s goals (i.e., to compete or to cooperate). At the brain level, we highlight the recruitment of networks associated with self–other integration, demonstrating a key overlap over right parietal areas.

### Attribution of Intention to a VP

At the behavioral level, our results first demonstrate how interaction with the HDC leads to the successful attribution of intention. Indeed, participants accurately judged the intention of the VP despite the confounding factor of task difficulty arising from the performance of in-phase versus anti-phase coordination (more difficult). The experimental protocol also led to attribution of humanness, although the partner was always a computational model. Participants judged the VP to be human 47.3% of the time. Our data show that the presence of a transition in VP behavior (i.e., from cooperation to competition or vice-versa) modulated the attribution of humanness to the VP (cf., Fig. 3) and that global joint coordination (i.e., RP stability) was correlated with the accurate attribution of intention to the VP. These behavioral results therefore confirm the ecological validity of HDC and demonstrate the influence of a VP’s behavior on human social cognition. Previous human–machine interaction paradigms using gaze pattern have shown how contingency and task modulate humanness attribution (Pfeiffer et al. 2011). Other psychosocial parameters such as gender similarity between the VP and the human participant can also impact subjective reports (Guadagno et al. 2007). Previous findings show how the HDC allows a new exploration of relational patterns during real-time sensorimotor coordination and how the latter modulates subjective judgment (Kelso et al. 2009; Dumas et al. 2014a) and emotional responses (Zhang et al. 2016). Building upon those results and combining the HDC with high-density EEG recording provides an opportunity to uncover the neural dynamics underlying a nonverbal Turing test during real-time social interaction.

### Coordination of Self- and Other-Neural Networks and the Role of the Right Parietal Cortex

At the brain level, source analysis reveals multiple brain networks involved in social coordination operating at different frequencies. Motor areas are recruited, as highlighted by a significant decrease of high-alpha/mu (10–13 Hz) power over contralateral and medial Rolandic regions during execution of movement compared with rest (Fig. 5*A*). This result is consistent with numerous reports of mu desynchronization during action execution (Salenius et al. 1997; Babiloni et al. 1999; Pfurtscheller et al. 2006; Dumas et al. 2014c; Hobson and Bishop 2016). Furthermore, in line with previous studies targeting reciprocal exchange of movement information during social interactions (Dumas et al. 2010; Novembre et al. 2016), the present EEG results exhibit decreases in high-alpha power over the superior aspect of right posterior parietal areas during social coordination in comparison to similar movement produced without a partner (Fig. 5*B*). The topography observed here is evocative of a body of work in monkey relating posterior parietal cortex to online control of visually guided movement (Buneo and Andersen 2006; McGuire and Sabes 2011)—with the caveat that caution is warranted due to task (individual goal-directed actions vs. social coordination) and species differences (monkey vs. human). Nevertheless, the present results are suggestive that posterior parietal alpha suppression reflects the visual coupling governing movement coordination between partners, whether virtual or not. Our data also expand current knowledge on parietal dynamics during on-line social interaction (Tognoli et al. 2007; Era et al. 2018a) by highlighting an increase of source-resolved gamma power over right temporo-parietal areas during social coordination compared with solo actions (Fig. 5*C*). Finally, interactions marked with high levels of emotional responses elicited increased alpha in a similar right parietal-temporal-insular complex (Fig. 5*D*; cf., Zhang et al. 2016). These results not only highlight the key role of right parietal areas in social coordination but also point toward a link between sensorimotor neuromarkers and affective dimensions of human social cognition.

Previous research has identified the involvement of right parietal cortex, specifically the TPJ for the integration of self and others’ actions (Sowden and Catmur 2015). TPJ is known to integrate inputs from subcortical (e.g., thalamus) and cortical (e.g., occipital, temporal, and prefrontal) regions (Decety and Lamm 2007). Functional neuroimaging studies have repeatedly linked activity over right TPJ (rTPJ) with socio-cognitive processes (Decety and Chaminade 2003), including theory of mind and empathy (Jackson et al. 2006), and joint-attention (Redcay et al. 2010). Moreover, research by Bzdok et al. (2013) has revealed the presence of two antagonistic clusters within rTPJ that is connected to networks processing external versus internal information. Reverse and forward inference meta-analyses linked the anterior cluster of rTPJ to “sensorimotor control […] integrating supramodal stimulus-guided attention and action initiation during externally structured tasks,” whereas the posterior cluster was linked to social cognition, theory of mind, and deception tasks. In our study, areas within the posterior rTPJ were part of a network significantly modulated by subjective judgments of humanness, and areas within the anterior rTPJ partook to a network affected by VP cooperation. Interfering with brain activity using Transcranial Magnetic Stimulation has shown a causal role of rTPJ in numerous processes relevant to social interaction, ranging from self-centered (i.e., body ownership; Tsakiris et al. 2008) and other-centered socio-cognitive processes (mentalizing and theory of mind; Bardi et al. 2017) to self–other integration in imitative actions (Sowden and Catmur 2015). Crucially, enhancement of rTPJ activity (using tDCS) improves online interactions by boosting the ability to switch between self- and other representations in both perspective-taking and the control of imitation (Santiesteban et al. 2012). Furthermore, Era et al. (2019) suggest that rTPJ supports the ability to perform joint movements only when self- and other-movement planning overlaps. The present research adds the understanding that rTPJ is the site of integration of corticomotor frequencies in the theta range for self and other, and that it is accompanied by higher frequencies in the gamma band for sensorimotor coordination.

Cortico-motor coherence in the theta band also reveals how right parietal sources are coordinated with shared movement velocity (Fig. 7), extending our previous results revealing a motor equivalence between the theta neural dynamics in motor cortex and velocity of self-generated movements (Kelso et al. 1998). The similar topography of the gamma increase, the alpha decrease, and the cortico-motor coherence in the theta band suggest a multifaceted integration of self- and other behaviors and a polyvalent role of right parietal areas in supporting sensorimotor coordination. While gamma increase of activity reflects local “processing,” alpha desynchronization has been previously linked to “gating” information (Varela et al. 1981; Busch and VanRullen 2010), in particular by routing information to task-relevant regions through inhibition of task-irrelevant ones (Jensen and Mazaheri 2010). Here, the joint increase of gamma and decrease of alpha in rTPJ was found to colocalize with the overlap of information about self- and other behavior, thus reflecting a change in the ongoing processing of self- and other-associated information. Activity in the theta band is usually associated with long-range connectivity and large-scale coordination of distant cortical areas (Moreau et al. 2018). Zhang et al. (2018) described theta waves as “traveling” waves, crucial for behavior by spatially and temporally organizing perceptual and cognitive processes across the cortex. In our case, we observed an increase of low-frequency connectivity across a large-scale network modulated by both sensorimotor and socio-cognitive factors, suggesting that the integration of socially relevant components during social interaction influences global cortical activity. Conversely, activity in the gamma band is often associated with the local processing of information (Fries 2009). Much previous research has reported a coupling of theta and gamma activity over similar areas thought to form a neural code for representing sequential order among numerous elements (Canolty and Knight 2010; Lisman and Jensen 2013). Here, the colocalization of the theta hub between self- and other network and the gamma activity associated with active social coordination points toward a functional cross-frequency link uniting the two processes.

Social interactions are not solely orchestrated by parietal regions. Moreau et al. (2018) showed an increase of occipito-temporal activity when a VP performed unexpected actions during motor interactions, highlighting the role of occipito-temporal areas in integrating the behavior of others. In accordance with this, we find that an increase of cortico-motor coherence over posterior sites is related to the partner’s movement (cf., Fig. 6). The present connectivity analysis revealed a significant increase of cortico-cortical coherence between bilateral occipital areas when participants declared they were interacting with a human partner (Fig. 8*C*,*E*).

### Brain Dynamics of Social Embodiment

Understanding the intentions of others is a crucial feature of effective social interaction. The present behavioral results highlight a correlation between sensorimotor performance and the correct attribution of intention. Furthermore, we show through whole-scalp connectivity analysis that large-scale connectivity modulation is associated with both high- (social cognition) and low-level (sensorimotor) aspects at play during live interaction. The attribution of human intentions to the VP is associated with higher coherence between bilateral occipital areas and between right occipital and parietal areas (coherent with the location of rTPJ) (cf., Fig. 8*A*,*C*,*E*), whereas cooperative behavior from the VP reveals larger right-lateralized fronto-parietal communication (including prefrontal, motor, right-parietal, right-temporal, and right-occipital areas) compared with competitive behavior (cf., Fig. 8*B*,*D*,*F*). These results confirm the spectral and the cortico-motor coherence findings, by emphasizing the presence large-scale brain networks related to the completion of our social task. Altogether, our results, showing the communication between visual and parietal areas, appear to fit the nexus model of rTPJ (Carter and Huettel 2013), which proposes that the visual processing of social information by occipital and occipito-temporal areas coordinates with the integration/segregation of relevant information in rTPJ. Crucially, both cooperative behavior and human attribution are associated with the increase of connectivity between posterior areas (occipital, temporal, and parietal) and motor and frontal areas, the latter generally recruited in decision-making tasks regardless of the presence of others (Tomlin et al. 2006; Campbell-Meiklejohn et al. 2017; Shaw et al. 2018; Thornton et al. 2019). Hence, our results serve to unpack the link between social cognition and communication and perceptive, cognitive, and social brain networks. Taken together, the present findings call for going beyond the social brain per se to embrace a more integrative perspective where sensorimotor abilities and accurate intention attribution are part and parcel of the same self-organizing coordination dynamics that grounds social awareness and cognition (Oullier and Kelso 2009; Kelso et al. 2013; Dumas et al. 2014b).

### Limitations

The current study presents several limitations. First, our sample size (*n* = 20) restricts statistical power. Another limitation concerns the use of EEG alone for studying the brain’s functional networks. EEG is a technique with an appropriately high-temporal resolution to conduct the key analyses, especially the cortico-motor coherence, but with a restricted spatial precision. Even though high-density EEG provides a better spatial resolution than regular EEG systems (e.g., 64 electrodes), volume conduction, a biophysical phenomenon defined as the transmission of electric fields from primary sources through biological tissues and recorded by several sensors at different locations (Wolters and de Munck 2007), distorts spatial precision. The use of a generic brain template instead of individual MRI also contributed to less accurate source estimation. Adding or combining MEG or fMRI is likely to reinforce the robustness of the study, and a priori powered sample selection will complement the current results and improve our understanding of the neural networks involved in reciprocal social interactions.

### Translational Potential and Conclusion

These limitations apart, the current results carry a large translational potential for neurological and psychiatric disorders. Ongoing and future studies use the HDC to investigate people on the autism spectrum (ASDs), to identify specific features affected in ASDs (i.e., ranging from sensorimotor to representational skills). The rationale behind this design is that deep phenotyping (Robinson 2012), from neural dynamics to social cognition, will help in understanding the heterogeneity of ASDs and identify intervention-relevant biomarkers. Furthermore, extending the findings of the current and previous studies of VP interaction might provide useful tools to help rehabilitation (e.g., patients recovering from strokes). For example, online interactions have shown promising results regarding motor performance in patients suffering from apraxia (Candidi et al. 2017). Altogether, the current results have both theoretical and practical implications, aiming to unite low-level and higher order frameworks of social interaction.

## Notes

We thank the Brainstorm Team for their software (http://neuroimage.usc.edu/brainstorm/) and G. de Guzman for help in implementing the human dynamic clamp. We also thank M. Zhang for her support in the earlier stages of this research.
